# Evaluating the Dose-Dependent Effects of Human Umbilical Cord-Derived Mesenchymal Stem Cells in a Preclinical Model of Interstitial Lung Disease

**DOI:** 10.3390/ijms262010016

**Published:** 2025-10-15

**Authors:** Takuya Kotani, Takashi Saito, Ryota Masutani, Satsuki Uemura, Shogo Matsuda, Takayasu Suzuka, Masaki Ikemoto, Tohru Takeuchi

**Affiliations:** 1 Department of Internal Medicine (IV), Division of Rheumatology, Osaka Medical and Pharmaceutical University, Takatsuki 569-8686, Osaka, Japan; takuya.kotani@ompu.ac.jp (T.K.);; 2 Department of Legal Medicine, Osaka Medical and Pharmaceutical University, Takatsuki 569-8686, Osaka, Japan; 3 Laboratory of Microbial Informatics, National Institutes of Biomedical Innovation, Health and Nutrition, Ibaraki 567-0085, Osaka, Japan; 4 Division of Central Laboratory, Osaka Medical and Pharmaceutical University, Takatsuki 569-8686, Osaka, Japan; 5 School of Medicine, Osaka Medical and Pharmaceutical University, Takatsuki 569-8686, Osaka, Japan

**Keywords:** connective tissue disease, human umbilical cord-derived mesenchymal stem cell, interstitial lung disease, MMP-9, TIMP-1

## Abstract

Interstitial lung disease associated with connective tissue disease (CTD-ILD) is a severe condition characterized by inflammation and progressive lung fibrosis, with limited treatment options. Previous studies have demonstrated the anti-inflammatory and antifibrotic properties of human umbilical cord-derived mesenchymal stem cells (huMSCs), suggesting their potential as novel therapeutic agents. Therefore, we investigated the dose-dependent therapeutic effects of huMSCs on CTD-ILD. A bleomycin-induced mouse model of interstitial lung disease, in which female C57BL/6J mice developed diffuse pulmonary lesions following continuous subcutaneous infusion of bleomycin, was used. Mice subsequently received intravenous huMSCs at doses of 1.0 × 10^3^, 1.0 × 10^4^, or 1.0 × 10^5^ cells. The medium dose (1.0 × 10^4^ cells) showed the most pronounced effects on pulmonary fibrosis and collagen deposition, while significantly suppressing pro-inflammatory cytokines, including interleukin-1β and interleukin-6; however, this effect was not consistent across all measured outcomes. The treatment also enhanced beneficial matrix remodeling by downregulating TIMP-1 and upregulating MMP-9 expression. Furthermore, huMSC administration modulated macrophage polarization and inhibited the pro-inflammatory M1 phenotype. These findings highlight the therapeutic potential of huMSCs for CTD-ILD and underscore the importance of dose optimization to balance efficacy and safety.

## 1. Introduction

Connective tissue disease-associated interstitial lung disease (CTD-ILD) is a severe complication that critically affects the prognosis of affected individuals [1]. Certain subtypes, such as progressive ILD linked to dermatomyositis and polymyositis, systemic sclerosis-associated progressive ILD, and acute ILD exacerbations in rheumatoid arthritis, are associated with poor survival outcomes and have major therapeutic challenges [2,3]. CTD-ILD management predominantly relies on corticosteroids in combination with immunosuppressive agents, such as calcineurin inhibitors, cyclophosphamide, and mycophenolate mofetil [4]. Despite these treatments, disease progression is often observed, which can lead to respiratory failure or death. Moreover, serious adverse events, including immunosuppression-related infections and drug-induced organ damage, frequently occur, thereby limiting their long-term use [5]. Antifibrotic medications, such as pirfenidone and nintedanib, have demonstrated potential in slowing fibrosis, but their therapeutic benefits remain unsatisfactory. Therefore, an urgent need to develop novel and effective treatments for progressive CTD-ILD is present [6,7].

Mesenchymal stem cells (MSCs) are increasingly recognized as valuable assets in cell-based therapy owing to their multipotency and significant immunomodulatory effects. Beyond their ability to differentiate into mesenchymal-derived tissues, including bone, adipose, and cartilage, MSCs also possess anti-inflammatory and antifibrotic capabilities, which they exert by influencing the local microenvironment at injury sites [6,8]. MSCs effectively suppress the activity of effector T cells and modulate innate immune responses by releasing bioactive molecules, such as interleukin-6 (IL-6) and prostaglandin E2, thereby reducing chronic inflammation [9,10]. Furthermore, MSCs contribute to tissue repair and mitigate fibrosis by secreting matrix metalloproteinases (MMPs) and hepatocyte growth factors, which are pivotal for tissue remodeling [8,11,12,13]. These distinctive characteristics render MSCs a compelling therapeutic candidate for challenging conditions, such as CTD-ILD, where they may have the potential to alleviate disease manifestations and enhance long-term outcomes.

Among the diverse sources of MSCs, umbilical cord-derived MSCs (uMSCs) have attracted considerable interest owing to their distinct advantages. First, umbilical cord blood is a non-invasive and ethically acceptable source of MSCs [14,15]. Second, uMSCs exhibit a greater capacity for proliferation than their counterparts derived from bone marrow or adipose tissue, allowing for efficient expansion even in patients with chronic illnesses [16,17]. Additionally, uMSCs demonstrate immunomodulatory functions, including the ability to suppress T and B cell activation, while promoting the generation of regulatory T cells, thereby mitigating chronic inflammatory responses [18,19,20].

We investigated the therapeutic potential of uMSCs in CTD-ILD using a bleomycin (BLM)-induced mouse model of interstitial lung disease. By exploring the immunomodulatory and antifibrotic capabilities of uMSCs, this study can contribute to their clinical application in managing CTD-ILD and improving patient outcomes.

## 2. Results

### 2.1. Therapeutic Effects of Human uMSCs on Pulmonary Fibrosis in ILD

**Figure 1:**
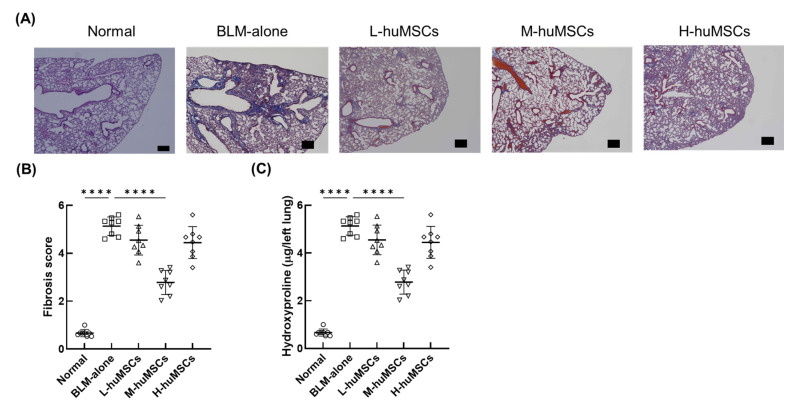
Therapeutic Impact of huMSCs on Pulmonary Damage in Mice 28 Days Post-BLM Administration. (**A**) Representative images highlighting areas of collagen deposition in lung tissue. Scale bars: 300 μm. (**B**) Quantitative fibrosis scoring for the different treatment groups. (**C**) Levels of hydroxyproline were measured in the lung tissues. The experimental groups include: Normal (untreated control mice), BLM-alone (mice with interstitial lung disease induced by BLM), L-huMSCs (low-dose huMSCs treatment, 1 × 10^3^ cells), M-huMSCs (medium-dose huMSCs treatment, 1 × 10^4^ cells), and H-huMSCs (high-dose huMSCs treatment, 1 × 10^5^ cells). Results are presented as the mean ± SD *(n* = 8 mice per group). Significant differences were shown between Normal and BLM-alone, as well as between BLM-alone and other treatment groups. Statistical significance: **** *p* < 0.0001 for comparisons between indicated groups. BLM, bleomycin; huMSC, human umbilical cord-derived mesenchymal stem cell.

Excessive collagen deposition resulted in fibrosis in lungs affected by BLM-induced ILD. The administration of human uMSCs (huMSCs) alleviated pulmonary fibrosis in a dose-dependent manner, with noticeable reductions observed at low and medium cell doses. However, this effect was not observed at higher doses (Figure 1A). The fibrosis score in the group treated with BLM alone was significantly higher than that in the control group. In contrast, it was significantly lower in the M-huMSC group than that in the BLM-alone group. In contrast, no significant differences in fibrosis scores were detected between the L-huMSC and H-huMSC groups and the BLM-alone group (Figure 1B). Collagen content was markedly elevated in the BLM alone group compared with that in the normal group, but was significantly reduced in the L-huMSC, M-huMSC, and H-huMSC groups when compared to the BLM alone group (Figure 1C). Notably, the collagen levels in the H-huMSC group were slightly higher than those in the L-huMSC and M-huMSC groups. Comparative analyses among the treatment groups highlighted a notable difference in fibrosis severity and collagen deposition between the intermediate and high doses (Figure S1). Additionally, the expression of the *MMP-9* showed a significant variation between the dosing groups (Figure S2). These results indicated that huMSCs possess hypoimmunogenic properties; however, excessive dosing may provoke huMSC-derived inflammation.

### 2.2. The huMSCs Regulated the Genes Associated with the Anti-Inflammatory and Antifibrotic Effects in ILD

**Figure 2:**
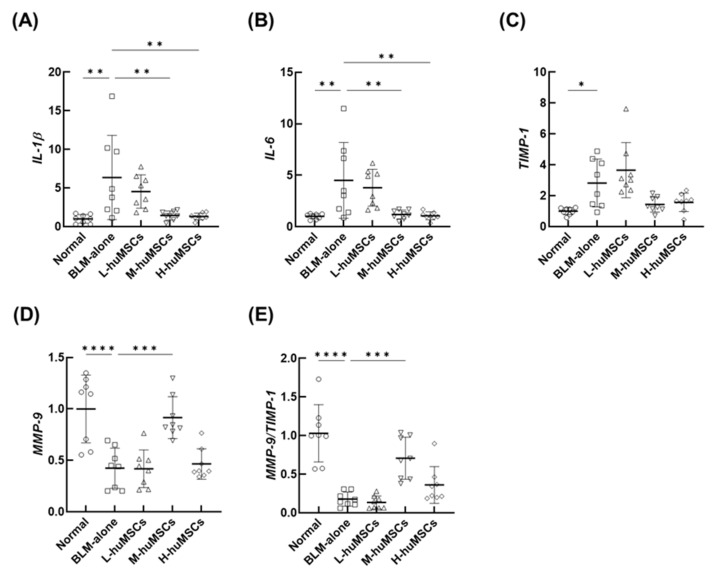
Quantitative Reverse Transcription-Polymerase Chain Reaction Analysis of Lung mRNA Expression. The relative mRNA expression of IL-1β (**A**), IL-6 (**B**), TIMP-1 (**C**), and MMP-9 (**D**) was evaluated in the lung tissues of BLM-induced ILD mice at day 28. Experimental groups included: Normal (untreated control mice), BLM-alone (mice with ILD induced by BLM), L-huMSCs (low-dose huMSCs, 1 × 10^3^ cells), M-huMSCs (medium-dose huMSCs, 1 × 10^4^ cells), and H-huMSCs (high-dose huMSCs, 1 × 10^5^ cells). Results are presented as the mean ± SD (*n* = 8 mice per group). Statistical significance is denoted as follows: * *p* < 0.05, ** *p* < 0.01, *** *p* < 0.001, **** *p* < 0.0001, with significant differences highlighted between the indicated groups. ns represents no significant difference. BLM, bleomycin; huMSC, human umbilical cord-derived mesenchymal stem cell; ILD, interstitial lung disease.

To investigate the anti-inflammatory and antifibrotic properties of huMSCs, the mRNA expression levels of the key inflammatory cytokines, MMP-9, and tissue inhibitor of MMP-1 (TIMP-1) in whole lung tissue were assessed using quantitative reverse transcription-polymerase chain reaction (qRT-PCR). The relative mRNA expression levels of IL-1β (Figure 2A) and IL-6 (Figure 2B) were significantly elevated in the BLM-alone group compared with those in the normal group. These levels were significantly reduced in a dose-dependent manner in the M-huMSC and H-huMSC groups compared with those in the BLM-alone group. Similarly, TIMP-1 expression was markedly higher in the BLM-alone group than in the normal group, and decreased in the M-huMSC and H-huMSC groups in a dose-dependent manner (Figure 2C). The relative mRNA expression levels of *MMP-9* and MMP-9/TIMP-1 ratio were significantly decreased in the BLM-alone group compared with those in the normal group, and significantly increased in the M-huMSC group compared with those in the BLM-alone group (Figure 2D,E).

### 2.3. Immunohistochemical Analysis of CD68-Positive Macrophages

**Figure 3:**
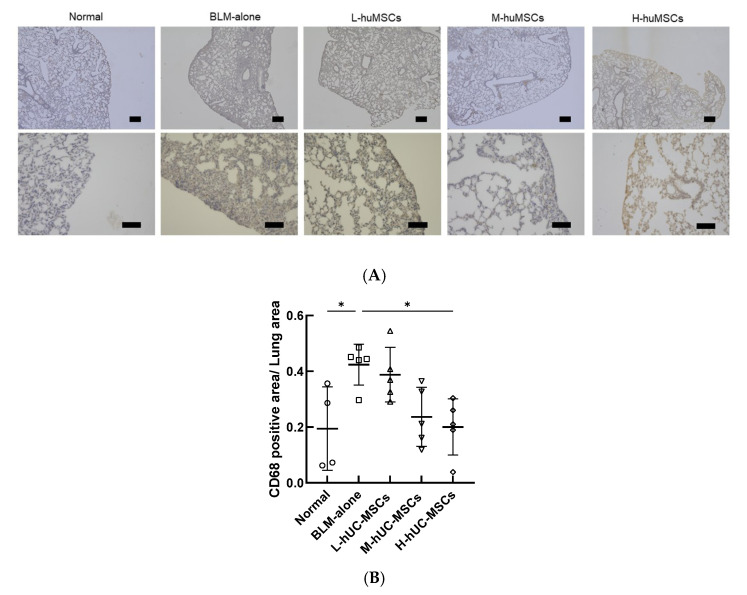
Immunohistochemical analysis of CD68-positive macrophages in lung sections. (**A**) Representative images of CD68 immunostaining in the Normal, BLM-alone, L-huMSC, M-huMSC, and H-huMSC groups. Brown-stained regions represent CD68-positive macrophages. Images were obtained at ×40 (scale bar: 300 μm) and ×200 (scale bar: 100 μm) magnification. (**B**) Quantification of CD68-positive area relative to total lung area. The ratio was significantly increased in the BLM-alone group compared with the Normal group, and significantly reduced in the H-huMSC group compared with the BLM-alone group. No significant differences were observed in the L-huMSC or M-huMSC groups. Data are presented as mean ± SD. * *p* < 0.05; ns, not significant.

Immunohistochemical analysis of CD68-positive macrophages revealed a significant increase in the CD68-positive area/total lung area ratio in the BLM-alone group compared with the Normal group. Among the treatment groups, the H-huMSC group showed a significant reduction in this ratio compared with the BLM-alone group. In contrast, no significant differences were observed in the L-huMSC or M-huMSC groups compared with the BLM-alone group. Representative immunohistochemical images and the quantitative analysis are demonstrated in Figure 3.

### 2.4. The huMSCs Suppressed the Polarization of Mouse Macrophages Toward M1 Dominance In Vitro

**Figure 4:**
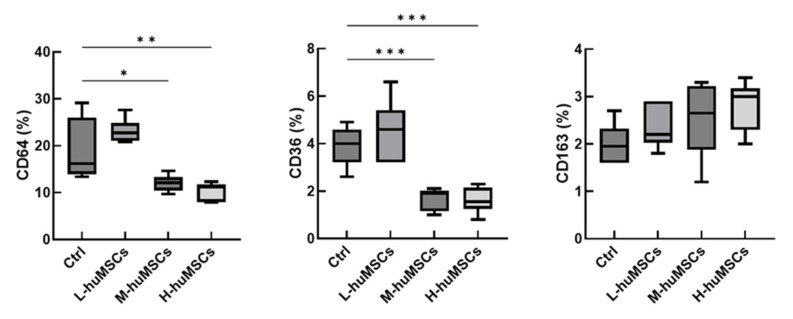
Surface Antigen Expression in Murine Macrophages. The expression levels of CD64, CD36, and CD163 were analyzed on the surface of murine macrophages. Experimental groups included L-huMSCs (co-cultured with 1 × 10^3^ huMSCs), M-huMSCs (co-cultured with 1 × 10^4^ huMSCs), H-huMSCs (co-cultured with 1 × 10^5^ huMSCs), and Ctrl (control group without huMSC co-culture). Results are expressed as mean ± SD (*n*= 6 per group). Statistical significance is indicated as follows: * *p* < 0.05, ** *p* < 0.01, *** *p* < 0.001, with significant differences highlighted between the specified groups. huMSC, human umbilical cord-derived mesenchymal stem cell.

**Figure 5:**
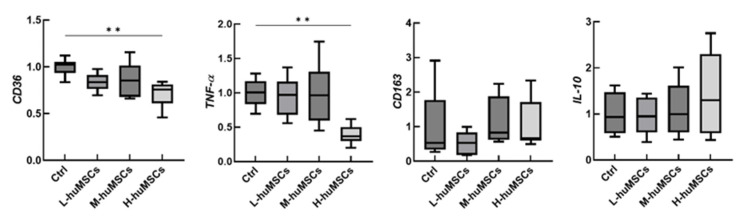
Gene Expression in Murine Macrophages. Relative mRNA expression of CD36, TNF-α, CD163, and IL-10 in murine macrophages. L-huMSCs: Co-cultured with a low dose of huMSCs (1 × 10^3^ cells) group. M-huMSCs: Co-cultured with a medium dose of huMSCs (1 × 10^4^ cells) group. H-huMSCs: Co-cultured with a high dose of huMSCs (1 × 10^5^ cells) group. Ctrl: Control group in which huMSCs were not co-cultured. Data are shown as mean ± SD (*n* = 6 per group). ** *p* < 0.01, significant differences between the linked groups. huMSC, human umbilical cord-derived mesenchymal stem cell.

The results of cell surface antigen analysis of murine macrophages are shown in Figure 4. The expression levels of CD64 and CD36 were significantly decreased in the huMSC co-culture group compared with those in the control group without huMSCs, exhibiting a dose-dependent relationship. In contrast, CD163 expression remained unchanged. Gene expression analysis further demonstrated that CD36 and TNF-α levels were markedly reduced in the huMSC co-culture group in a cell dose-dependent manner compared with those in the control group, whereas no significant changes were observed in CD163 or IL-10 expression levels (Figure 5). These findings suggest that co-culture with huMSCs may suppress M1-dominant polarization in murine macrophages.

## 3. Discussion

We investigated the therapeutic potential of huMSCs in treating pulmonary fibrosis in a mouse model of BLM-induced interstitial lung disease mouse model. These findings revealed a dose-dependent effect, with the intermediate dose achieving the most significant reduction in fibrosis and collagen deposition (Figure 1). Moreover, huMSCs influenced key inflammatory and fibrotic pathways, as demonstrated by decreased levels of *IL-1β*, *IL-6*, and *TIMP-1,* alongside increased *MMP-9* expression (Figure 2).

In vitro findings further demonstrated their capacity to inhibit M1 macrophage polarization by suppressing pro-inflammatory markers, such as CD36 (intermediate dose) and TNF-α (intermediate and high dose) (Figure 4). Collectively, these observations emphasize the dual immunomodulatory and antifibrotic functions of huMSCs, reinforcing their potential as a therapeutic strategy for interstitial lung disease.

The therapeutic effects of huMSCs in preclinical ILD trials, comparing previous findings with those of the current study, are summarized in Table 1 [21,22,23,24,25,26,27,28]. Previous studies, as well as the present study, have reported that huMSCs mitigated fibrosis progression and reduced inflammation in pulmonary disease models. Earlier reports have primarily shown reductions in lung collagen content, improved histopathological scores, and suppressed inflammatory cytokine levels, consistent with our results. Notably, the observed increase in MMP-9 and decrease in TIMP-1 levels were consistent with those of previous studies, reinforcing their roles in fibrosis suppression.

Macrophage polarization from the M1 to M2 phenotype has been a key area of investigation. Meng et al. [28] have highlighted the role of huMSCs in inhibiting M1 polarization to exert anti-inflammatory effects. Our findings support this hypothesis by demonstrating that huMSCs facilitate macrophage polarization, suppress pro-inflammatory conditions, and mitigate fibrosis progression.

A major strength of this study is its thorough assessment of the huMSC dose–response relationship, which enabled the identification of an optimal therapeutic dose. Chu et al. used a rat model of BLM-induced pulmonary fibrosis model in rats [25]. The huMSCs were intratracheally administered at doses of 5 × 10^6^ and 2.5 × 10^7^ cells, which served as a reference for dose selection in this study. They reported significant improvements in lung function, reduced collagen deposition, and increased MMP-9 expression in the high-dose group, whereas the low-dose group exhibited only modest effects, highlighting the benefits of higher doses for established pulmonary fibrosis. In contrast, in this study, we used a BLM-induced pulmonary fibrosis model in mice and investigated the intravenous administration of huMSCs at doses of 1.0 × 10^3^, 1.0 × 10^4^, and 1.0 × 10^5^ cells. The results demonstrated that the medium dose provided the most pronounced therapeutic benefits, including suppression of inflammatory cytokines (IL-6 and IL-1β), enhanced MMP-9 expression, and the highest reduction in fibrosis scores, compared with those of the other doses. The high-dose group exhibited reduced efficacy, indicating that excessive cell delivery may disrupt the lung microenvironment or hinder the therapeutic effects.

The differences in therapeutic outcomes between this study and those reported by Chu et al. may be attributed to differences in model organisms (rats in their study and mice in this study) as well as the route of huMSC administration [26]. Chu et al. intratracheally delivered cells, potentially resulting in higher local cell concentrations at the site of injury, whereas intravenous administration was used in the present study, potentially resulting in broader cell distribution. These variations in delivery routes affect the biodistribution and pharmacokinetics of huMSCs, thereby altering local cellular interactions, which may explain the differences in therapeutic efficacy. These findings highlight the necessity of carefully selecting appropriate models and administration routes in preclinical research, and emphasize the necessity of precise dose optimization to optimize efficacy, while ensuring safety in huMSC-based therapies for pulmonary fibrosis.

The results suggest that high-dose huMSC administration may not be optimal for improving fibrosis. This may be due to a transient activation of the immune system caused by an excessive number of cells, partially counteracting the anti-inflammatory effects observed with the intermediate dose. MSCs have the ability to secrete immunosuppressive factors in response to the inflammatory environment, thereby modulating immune responses [29]. However, an excessive cell number can alter the local immune milieu and, paradoxically, enhance immune activation. Therefore, in MSC therapy, optimizing the dose to appropriately modulate immune responses and maximize therapeutic efficacy is crucial.

Our findings revealed that the therapeutic effects of high-dose huMSCs were inferior to those observed in the medium-dose group and, in some cases, may exacerbate pulmonary fibrosis. This finding is consistent with previous research indicating that, despite their recognized immunosuppressive properties, MSCs can provoke immune responses, particularly in the context of xenotransplantation [30,31,32]. For example, xenotransplantation of huMSCs into mice has been associated with significant leukocyte infiltration at the injection site, which is indicative of the activation of the innate immune system activation [30]. Immune responses are reported to be most pronounced in xenogeneic transplantation, followed by allogeneic and syngeneic approaches [31,32]. These findings underscore the challenges in translating human MSC therapies into clinical practice, especially given the frequent reliance on xenotransplantation models for preclinical evaluation.

This study had some limitations. First, although the BLM-induced pulmonary fibrosis model is widely used, it does not fully capture the chronic and heterogeneous characteristics of CTD-ILD in humans. Second, the immune response, particularly in the high-dose huMSC group, may have been influenced by the xenotransplantation environment, potentially diminishing the therapeutic benefits to the cells. Previous studies have suggested the use of immunosuppressive agents, such as dexamethasone or tacrolimus, to mitigate immune activation in similar settings [30,33]. The integration of these immunosuppressive strategies into experimental models may enhance the dose-dependent efficacy of huMSCs. Third, only female mice were used as connective tissue diseases, including CTD-ILD, which predominantly affects women. However, the absence of male mice represents a limitation, as sex-related biological differences may influence inflammatory and fibrotic responses. Fourth, blood samples were not collected in this study, which precluded the assessment of circulating inflammatory markers. Future studies incorporating blood-based analyses will be important to complement histological findings and strengthen the overall evaluation of huMSC efficacy. Finally, this study was limited to short-term outcomes and investigated only intravenous administration, leaving the long-term effects and alternative delivery routes unexplored. Addressing these limitations in future studies is critical for the clinical advancement of huMSC therapy for CTD-ILD.

## 4. Materials and Methods

### 4.1. Ethics

All animal experiments were approved by the Animal Experimentation Committee of Osaka Medical and Pharmaceutical University (Approval ID: AM23-010), and they were conducted in accordance with the ARRIVE guidelines.

### 4.2. Materials

Dulbecco’s phosphate-buffered saline (PBS) was obtained from Nissui Pharmaceutical Co., Ltd. (Tokyo, Japan). Iscove’s Modified Dulbecco’s Medium (IMDM) GlutaMAX, 4% paraformaldehyde in PBS, and basic fibroblast growth factor (bFGF) were purchased from FUJIFILM Wako Pure Chemical Corporation (Osaka, Japan). Fetal bovine serum (FBS) was obtained from Biowest (Nîmes, France), and penicillin–streptomycin solution (P/S; 10,000 U/mL penicillin and 10 mg/mL streptomycin) was purchased from Merck KGaA (Darmstadt, Germany). Thioglycolate was obtained from Sigma-Aldrich (St. Louis, MO, USA), and Cell Counting Reagent SF from Nacalai Tesque, Inc. (Kyoto, Japan).

### 4.3. huMSCs

huMSCs were purchased from PromoCell GmbH (Heidelberg, Germany) (Lot No. 4502018). Cells were cultured at 37 °C under 5% CO_2_ in IMDM supplemented with 20% FBS, 2 mM L-glutamine, 10 ng/mL bFGF, and 1.0% P/S for 24 h. Passage 4 huMSCs were used for in vivo experiments, whereas passage 5 cells were used in vitro.

### 4.4. Animal Models and Surgical Procedure

Thirteen-week-old female C57BL/6J mice (Shimizu Laboratory Supplies Co., Ltd., Kyoto, Japan) were anesthetized with 5% isoflurane for 3 min and maintained at 1.5–2.0% during procedures. Mice were randomly assigned to five groups: a normal group with no treatment; a BLM group with BLM-induced interstitial lung disease (ILD); an L-huMSC group with BLM plus 1.0 × 10^3^ huMSCs; an M-huMSC group with BLM plus 1.0 × 10^4^ huMSCs; and an H-huMSC group with BLM plus 1.0 × 10^5^ huMSCs. Each group included eight mice. 

Bleomycin sulfate (Nippon Kayaku Co., Ltd., Tokyo, Japan) was dissolved in sterile saline, and 100 μL (3 mg) was loaded into an osmotic mini-pump (Alzet; DURECT, Cupertino, CA, USA), which was implanted subcutaneously in the dorsal region under anesthesia. Continuous subcutaneous BLM administration induces inflammation and fibrosis around the pleura and vessels, closely resembling lesions observed in patients with CTD-ILD [34]. Cell dose selection was guided by findings from preliminary experiments in which administration of 1.0 × 10^4^ huMSCs demonstrated a trend toward therapeutic benefit. To systematically assess dose–response relationships, one lower dose (1.0 × 10^3^) and one higher dose (1.0 × 10^5^) were additionally included.

On Day 7, huMSCs were suspended in 100 μL PBS and administered intravenously via the tail vein. Negative control mice received 100 μL PBS. Mice were euthanized 21 days after cell administration under 5% isoflurane anesthesia via cervical dislocation, and lungs were collected. The total observation period was 28 days. To systematically assess dose–response relationships, one lower dose (1.0 × 10^3^) and one higher dose (1.0 × 10^5^) were additionally included, based on preliminary experiments in which administration of 1.0 × 10^4^ huMSCs demonstrated a trend toward therapeutic benefit. 

### 4.5. Preparation of Mouse Macrophages and Co-Culture with huMSCs

Mouse macrophages were obtained 4 days after intraperitoneal injection of 1.0 mL 4% thioglycolate in PBS under anesthesia. Peritoneal cells were collected with 5 mL ice-cold PBS, centrifuged at 1000 rpm for 10 min at 4 °C, resuspended in RPMI-1640 supplemented with 10% FBS, and counted.

For co-culture, huMSCs (1.0 × 10^3^, 1.0 × 10^4^, or 1.0 × 10^5^ cells) were seeded in six-well plates with IMDM containing 1.0% FBS, 2 mM L-glutamine, 10 ng/mL bFGF, and 1.0% P/S. Macrophages were added and co-cultured for 24 h. Adherent cells were washed three times with PBS, detached using a scraper, collected in 1 mL RPMI-1640 with 10% FBS, centrifuged at 1000 rpm for 10 min at 4 °C, and supernatants discarded.

### 4.6. Flow Cytometry

Macrophages were resuspended in fluorescence-activated cell sorting (FACS) buffer (BD Biosciences, Franklin Lakes, NJ, USA) and passed through a 40 µm filter. For each sample, 3.0 × 10^5^ cells were incubated with 10 µL of PE-Cyanine7-conjugated anti-mouse CD64 (1:100), PerCP/Cyanine5.5-conjugated anti-mouse CD36 (1:100), and APC-conjugated anti-mouse CD163 (1:100) antibodies (BioLegend, San Diego, CA, USA) for 30 min at 4 °C in the dark. Cells were washed twice with FACS buffer, and 10,000 events were acquired per sample using a Navios flow cytometer (Beckman Coulter, Brea, CA, USA). Live cells were gated based on FSC/SSC.

### 4.7. In Vitro qRT-PCR of Macrophages

Total RNA was extracted using the RNeasy Mini Kit (Qiagen, Hilden, Germany), treated with DNase, and reverse-transcribed into cDNA using the ExScript RT kit (Takara, Shiga, Japan). Quantitative PCR was performed on an ABI PRISM 7000 Sequence Detection System (Applied Biosystems, Tokyo, Japan). Primers (Supplementary Table S1) targeted CD36, CD163, TNF-α, and IL-10; GAPDH served as the housekeeping gene. Expression levels were calculated using the ΔΔCT method. Each experiment was repeated four times independently, with samples analyzed in triplicate.

### 4.8. Histology and Fibrosis Assessment

The right middle lung lobe was fixed in 4% PFA/PBS for 6 h and then immersed in 20% sucrose/PBS overnight. Paraffin sections (5 µm) were stained with Masson’s trichrome. Five non-overlapping fields showing the most severe fibrosis were selected (×200 magnification). Fibrosis was scored using a modified Ashcroft scale (0–8) by three independent observers [35].

### 4.9. Hydroxyproline Assay

The left lung was stored at −70 °C, hydrolyzed in 0.5 M acetic acid with pepsin (0.3 mg/10 mg tissue) at 4 °C for 18 h, neutralized, and the collagen content was measured using the Sircol™ Soluble Collagen Assay Kit (Biocolor Ltd., Belfast, UK). Dye release was quantified at 556 nm using a microplate reader.

### 4.10. In Vivo Lung qRT-PCR

Lung RNA was extracted 28 days after cell administration using NucleoSpin^®^ RNA (MACHEREY-NAGEL, Düren, Germany). Targets were IL-6, IL-1β, MMP-9, and TIMP-1; GAPDH served as the housekeeping gene (Supplementary Table S2). PCR: 95 °C 3 min, then 40 cycles of 95 °C 10 s and 60 °C 30 s. Expression was calculated using ΔΔCT, normalized to GAPDH. Experiments were performed in triplicate.

### 4.11. Immunohistochemical Staining

Immunohistochemical staining of lung tissues was performed as described previously [36]. Lung tissues fixed in 10% formalin were embedded in paraffin, sectioned at 4 µm, and mounted on APS-coated slides. After deparaffinization and rehydration, antigen retrieval was carried out in 20 mM citrate buffer (pH 6.0). Endogenous peroxidase and biotin activities were blocked using 0.3% hydrogen peroxide/methanol and HISTOFINE (NICHIREI BIOSCIENCE INC., Tokyo, Japan), respectively. Sections were blocked with 1.6% goat serum and incubated overnight at 4 °C with anti-CD68 (ED1)-biotin antibody (GTX43915, GeneTex, Funakoshi Co. Ltd., Tokyo, Japan). Detection was performed using streptavidin–horseradish peroxidase conjugate (SA-5004, Vector Laboratories, Burlingame, CA, USA) and 3,3’-diaminobenzidine, followed by hematoxylin counterstaining. Positive macrophages were observed under a microscope (SH-1000Lab, Corona Electric Co., Ltd., Ibaraki, Japan).

### 4.12. Immunohistochemical Analysis of CD68-Positive Macrophages

Immunohistochemical staining for CD68 was performed on lung tissue sections to identify macrophages. The brown-stained regions were defined as CD68-positive areas. For each section, five non-overlapping fields showing the most severe fibrosis were selected at ×200 magnification. The CD68-positive area and the total lung tissue area within each field were measured using a WinROOF2021 computerized morphometry system (Mitani, Fukui, Japan). The mean ratio of CD68-positive area to total lung tissue area across the five fields was calculated for each mouse, and these values were compared among the different treatment groups (Normal group, *n* = 4; BLM-alone and each treatment group, *n* = 5).

### 4.13. Statistical Analysis

Data are expressed as mean ± SD. Analyses were performed using GraphPad Prism 9.5.1 (GraphPad Software, San Diego, CA, USA). One-way ANOVA with Tukey’s post hoc test was used for multiple-group comparisons. Mann–Whitney U test was applied for two-group comparisons. *p* < 0.05 was considered statistically significant.

## 5. Conclusions

In conclusion, our findings revealed that huMSCs have substantial potential for treating pulmonary fibrosis through dose-dependent anti-inflammatory and antifibrotic mechanisms. The identification of a medium dose as the most effective treatment highlights the importance of optimizing therapeutic dosing to maximize efficacy while minimizing potential adverse effects.

## Figures and Tables

**Table 1:** Therapeutic effects of human umbilical cord-derived mesenchymal stem cells (huMSCs) on experimental models of interstitial lung disease.

Author, Year [References]	Animal	Cell Source and Treatment	BLM Dose	Number of Injected Cells (×10^6^)	Transplant Time After Modeling (Route)	Outcome Time	Major Treatment Effects
Min, 2015 [21]	C57BL/6 mice	huMSCs	40 mg/kg	ND	0 d (IV)	7, 14, 28 d	↓ Lung collagen, ↓ IL-1β, ↓ IL-6, ↑ MMP-9
Moradi, 2017 [22]	6–8 w C57BL/6 mice	huMSCs	2 mg/kg	0.5	15 min (IT)	21 d	↓ Lung collagen, ↓ Ashcroft score, NS in weight, ↓ IL-1, ↓ IL-6
Moroncini, 2018 [23]	12–16 w C57BL/6 mice	huMSCs	1.5 mg/kg	0.25	24 h and 7 d (IV)	8, 14, 21 d	↓ Ashcroft score, ↓ IL-1β, ↓ IL-6, ↓ TIMP-1
Orlando, 2019 [24]	12–16 w C57BL/6 mice	huMSCs	1.5 mg/kg	0.25	24 h and 7 d (IV)	8, 14, 21 d	↓ Lung collagen, ↓ Soluble collagen, ↑ Weight
Chu, 2019 [25]	SD rats	huMSCs	8 mg/kg	5, 25	21 d (IT)	49 d	↓ Lung collagen, ↑ IL-6, ↑ MMP-9
Chu, 2020 [26]	8 w SD rats	huMSCs	5 mg/rat	25	21 d (IT)	49 d	↓ Lung collagen, ↑ Weight, ↓ IL-1, ↓ IL-6, ↑ MMP-9, NS in αSMA
Xian, 2022 [27]	7-w C57BL/6 male mice	huMSCs and pirfenidone (30 mg/kg)	3 mg/kg	0.5	7 d (IV)	21 d	↓ Lung collagen, ↓ Ashcroft score, ↑ Survival,↓ Fibrosis markers (Col1a1, Col1a2, α-SMA), ↑ RGS2 expression
Meng, 2024 [28]	6–8 w C57BL/6 mice	huMSCs	2 mg/kg	1	3, 9, 15 d (IV)	21, 29 d	↓ Lung collagen, ↓ Ashcroft score, ↑ Lung function, ↓ Inflammatory cytokines, ↓ M1 macrophages, ↑ M2 macrophages
Current Study	13-w C57BL/6 mice	huMSCs	3 mg/mouse	0.001 (L), 0.01 (M), 0.1 (H)	7 d (IV)	28 d	↓ Lung collagen, ↓ Ashcroft score,↓ IL-1β, ↓ IL-6, ↓ TIMP-1, ↑ MMP-9, ↓ M1 macrophage polarization

BLM: Bleomycin, hUC-MSCs: Human umbilical cord mesenchymal stem cells, IV: Intravenous, IT: Intratracheal, SD rats: Sprague–Dawley rats, L: a low dose of hUC-MSCs, M: a medium dose of hUC-MSCs, H: a high dose of hUC-MSCs, TIMP-1: Tissue inhibitor of metalloproteinase-1, MMP-9: Matrix metalloproteinase-9, IL-1β: Interleukin-1 beta, IL-6: Interleukin-6, TNF-α: Tumor necrosis factor alpha, Col1α1: Collagen 1 alpha 1, Col1α2: Collagen 1 alpha 2, αSMA: alpha-smooth muscle actin, RGS2: regulator of G protein signaling 2, ND: Not described, NS: Not significant, ↑: Increased, ↓: Decreased, d: Days, w: Weeks, g: Grams.

## Data Availability

No new datasets were generated or analyzed in this study. All experiments were performed using cell cultures and animal models. For data inquiries, please contact the corresponding author.

## Supplementary Materials

The following supporting information can be downloaded at: https://www.mdpi.com/article/10.3390/ijms262010016/s1.

## Abbreviations

The following abbreviations are used in this manuscript:

bFGF	Basic fibroblast growth factor
BLM	Bleomycin
CTD	Connective tissue disease
FBS	Fetal bovine serum
huMSC	Human umbilical cord-derived mesenchymal stem cell
ILD	Interstitial lung disease
IMDM	Iscove’s Modified Dulbecco’s Medium
P/S	Penicillin/streptomycin
PBS	Phosphate-buffered saline
uMSC	Umbilical cord-derived mesenchymal stem cell

## References

[1] Fischer A., du Bois R. (2012). Interstitial lung disease in connective tissue disorders. Lancet.

[2] Jeganathan N., Sathananthan M. (2020). Connective tissue disease-related interstitial lung disease: Prevalence, patterns, predictors, prognosis, and treatment. Lung.

[3] Vacchi C., Sebastiani M., Cassone G., Cerri S., Della Casa G., Salvarani C., Manfredi A. (2020). Therapeutic options for the treatment of interstitial lung disease related to connective tissue diseases: A narrative review. J. Clin. Med..

[4] Tashkin D.P., Elashoff R., Clements P.J., Goldin J., Roth M.D., Furst D.E., Arriola E., Silver R., Strange C., Bolster M. (2006). Cyclophosphamide versus placebo in scleroderma lung disease. N. E. J. Med..

[5] Yang M., Wu Y., Liu X., Zhao C., Li T., Li T., Zhang X., Jiang H., Mao B., Liu W. (2023). Efficacy and safety of antifibrotic agents in the treatment of CTD-ILD and RA-ILD: A systematic review and meta-analysis. Respir. Med..

[6] Murphy M.B., Moncivais K., Caplan A.I. (2013). Mesenchymal stem cells: Environmentally responsive therapeutics for regenerative medicine. Exp. Mol. Med..

[7] Qin L., Liu N., Bao C.L., Yang D.Z., Ma G.X., Yi W.H., Xiao G.Z., Cao H.L. (2023). Mesenchymal stem cells in fibrotic diseases—The two sides of the same coin. Acta Pharmacol. Sin..

[8] Maumus M., Jorgensen C., Noel D. (2013). Mesenchymal stem cells in regenerative medicine applied to rheumatic diseases: Role of secretome and exosomes. Biochimie.

[9] Di Nicola M., Carlo-Stella C., Magni M., Milanesi M., Longoni P.D., Matteucci P., Grisanti S., Gianni A.M. (2002). Human bone marrow stromal cells suppress T-lymphocyte proliferation induced by cellular or nonspecific mitogenic stimuli. Blood.

[10] Nauta A.J., Fibbe W.E. (2007). Immunomodulatory properties of mesenchymal stromal cells. Blood.

[11] Matsuda S., Kotani T., Saito T., Suzuka T., Mori T., Takeuchi T. (2021). Low-molecular-weight heparin enhanced therapeutic effects of human adipose-derived stem cell administration in a mouse model of lupus nephritis. Front. Immunol..

[12] Saito T., Kotani T., Suzuka T., Matsuda S., Takeuchi T., Sato T. (2022). Adipose-derived stem/stromal cells with heparin-enhanced anti-inflammatory and antifibrotic effects mitigate induced pulmonary fibrosis in mice. Biochem. Biophys. Res. Commun..

[13] Suzuka T., Kotani T., Saito T., Matsuda S., Sato T., Takeuchi T. (2022). Therapeutic effects of adipose-derived mesenchymal stem/stromal cells with enhanced migration ability and hepatocyte growth factor secretion by low-molecular-weight heparin treatment in bleomycin-induced mouse models of systemic sclerosis. Arthritis Res. Ther..

[14] Nagamura-Inoue T., He H. (2014). Umbilical cord-derived mesenchymal stem cells: Their advantages and potential clinical utility. World J. Stem Cells.

[15] McElreavey K.D., Irvine A.I., Ennis K.T., McLean W.H. (1991). Isolation, culture and characterisation of fibroblast-like cells derived from the Wharton’s jelly portion of human umbilical cord. Biochem. Soc. Trans..

[16] Tan K., Zheng K., Li D., Lu H., Wang S., Sun X. (2017). Impact of adipose tissue or umbilical cord derived mesenchymal stem cells on the immunogenicity of human cord blood derived endothelial progenitor cells. PLoS ONE.

[17] Mitchell K.E., Weiss M.L., Mitchell B.M., Martin P., Davis D., Morales L., Helwig B., Beerenstrauch M., Abou-Easa K., Hildreth T. (2003). Matrix cells from Wharton’s jelly form neurons and glia. Stem Cells.

[18] Liang J., Zhang H., Hua B., Wang H., Lu L., Shi S., Hou Y., Zeng X., Gilkeson G.S., Sun L. (2010). Allogenic mesenchymal stem cells transplantation in refractory systemic lupus erythematosus: A pilot clinical study. Ann. Rheum. Dis..

[19] Chang J.W., Hung S.P., Wu H.H., Wu W.M., Yang A.H., Tsai H.L., Yang L.Y., Lee O.K. (2011). Therapeutic effects of umbilical cord blood-derived mesenchymal stem cell transplantation in experimental lupus nephritis. Cell Transplant..

[20] Tipnis S., Viswanathan C., Majumdar A.S. (2010). Immunosuppressive properties of human umbilical cord-derived mesenchymal stem cells: Role of B7-H1 and IDO. Immunol. Cell Biol..

[21] Min F., Gao F., Li Q., Liu Z. (2015). Therapeutic effect of human umbilical cord mesenchymal stem cells modified by angiotensin-converting enzyme 2 gene on bleomycin-induced lung fibrosis injury. Mol. Med. Rep..

[22] Moradi M., Rezaee M.A., Mohammadi M., Rezaie M.J., Jalili A., Rahmani M.R. (2017). Attenuating effect of long-term culture of umbilical cord vein mesenchymal stromal cells on pulmonary fibrosis in C57BL/6 mice. Iran. J. Allergy Asthma Immunol..

[23] Moroncini G., Paolini C., Orlando F., Capelli C., Grieco A., Tonnini C., Agarbati S., Mondini E., Saccomanno S., Goteri G. (2018). Mesenchymal stromal cells from human umbilical cord prevent the development of lung fibrosis in immunocompetent mice. PLoS ONE.

[24] Orlando F., Paolini C., Agarbati S., Tonnini C., Grieco A., Capelli C., Introna M., Provinciali M., Gabrielli A., Moroncini G. (2019). Induction of mouse lung injury by endotracheal injection of bleomycin. J. Vis. Exp..

[25] Chu K.A., Wang S.Y., Yeh C.C., Fu T.W., Fu Y.Y., Ko T.L., Chiu M.M., Chen T.H., Tsai P.J., Fu Y.S. (2019). Reversal of bleomycin-induced rat pulmonary fibrosis by a xenograft of human umbilical mesenchymal stem cells from Wharton’s jelly. Theranostics.

[26] Chu K.A., Yeh C.C., Kuo F.H., Lin W.R., Hsu C.W., Chen T.H., Fu Y.S. (2020). Comparison of reversal of rat pulmonary fibrosis of nintedanib, pirfenidone, and human umbilical mesenchymal stem cells from Wharton’s jelly. Stem Cell Res. Ther..

[27] Wu X., Gou H., Zhou O., Qiu H., Liu H., Fu Z., Chen L. (2022). Human umbilical cord mesenchymal stem cells combined with pirfenidone upregulates the expression of RGS2 in the pulmonary fibrosis in mice. Respir. Res..

[28] Li M., Li J., Wang Y., Jiang G., Jiang H., Li M., Zhu Z., Ren F., Wang Y., Yan M. (2024). Umbilical cord-derived mesenchymal stem cells preferentially modulate macrophages to alleviate pulmonary fibrosis. Stem Cell Res. Ther..

[29] Han X., Liao R., Li X., Zhang C., Huo S., Qin L., Xiong Y., He T., Xiao G., Zhang T. (2025). Mesenchymal stem cells in treating human diseases: Molecular mechanisms and clinical studies. Signal Transduct. Target. Ther..

[30] Hwang J.W., Myeong S.H., Lee N.H., Kim H., Son H.J., Chang J.W., Lee N.K., Na D.L. (2021). Immunosuppressant drugs mitigate immune responses generated by human mesenchymal stem cells transplanted into the mouse parenchyma. Cell Transplant..

[31] Norte-Munoz M., Gallego-Ortega A., Lucas-Ruiz F., Gonzalez-Riquelme M.J., Changa-Espinoza Y.I., Galindo-Romero C., Ponsaerts P., Vidal-Sanz M., Garcia-Bernal D., Agudo-Barriuso M. (2022). Immune recognition of syngeneic, allogeneic and xenogeneic stromal cell transplants in healthy retinas. Stem Cell Res. Ther..

[32] Hwang J.W., Lee N.K., Yang J.H., Son H.J., Bang S.I., Chang J.W., Na D.L. (2020). A comparison of immune responses exerted following syngeneic, allogeneic, and xenogeneic transplantation of mesenchymal stem cells into the mouse brain. Int. J. Mol. Sci..

[33] Diehl R., Ferrara F., Muller C., Dreyer A.Y., McLeod D.D., Fricke S., Boltze J. (2017). Immunosuppression for in vivo research: State-of-the-art protocols and experimental approaches. Cell Mol. Immunol..

[34] Harrison J.H., Lazo J.S. (1987). High dose continuous infusion of bleomycin in mice: A new model for drug-induced pulmonary fibrosis. J. Pharmacol. Exp. Ther..

[35] Hubner R.H., Gitter W., El Mokhtari N.E., Mathiak M., Both M., Bolte H., Freitag-Wolf S., Bewig B. (2008). Standardized quantification of pulmonary fibrosis in histological samples. Biotechniques.

[36] Koike A., Arai S., Yamada S., Nagae A., Saita N., Itoh H., Uemoto S., Totani M., Ikemoto M. (2012). Dynamic mobility of immunological cells expressing S100A8 and S100A9 in vivo: A variety of functional roles of the two proteins as regulators in acute inflammatory reaction. Inflammation.

