# Supplementary material for: Evaluating the Dose-Dependent Effects of Human Umbilical Cord-Derived Mesenchymal Stem Cells in a Preclinical Model of Interstitial Lung Disease
Source: Int J Mol Sci. 2025 Oct 15;26(20):10016. doi: 10.3390/ijms262010016 (PMC12564416; doi:10.3390/ijms262010016)
Supplement: Supplementary file 1 [file ijms-26-10016-s001.zip › ijms-3777293-supplementary.pdf]

Supplementary Materials

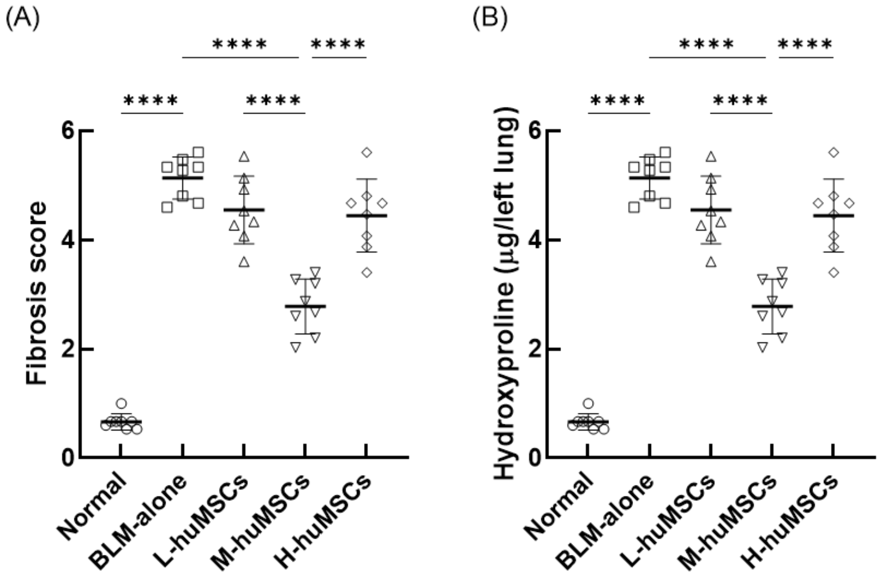

Figure S1. Therapeutic impact of huMSCs on pulmonary damage in mice 28 days post-bleomycin administration. **(A)** Quantitative fibrosis scoring for the different treatment groups. **(B)** Levels of hydroxyproline measured in the lung tissues. The experimental groups include: Normal (untreated control mice), BLM-alone (mice with interstitial lung disease induced by bleomycin), L-huMSCs (low-dose huMSCs treatment, 1×10^3 cells), M-huMSCs (medium-dose huMSCs treatment, 1×10^4 cells), and H-huMSCs (high-dose huMSCs treatment, 1×10^5 cells). Results are presented as the mean ± SD (N = 8 mice per group). Significant differences were displayed among Normal and BLM-alone, BLM-alone and the treatment groups, as well as between the treatment groups. Statistical significance: **** $p < 0.0001$ for comparisons between indicated groups; ns indicates no significant difference.

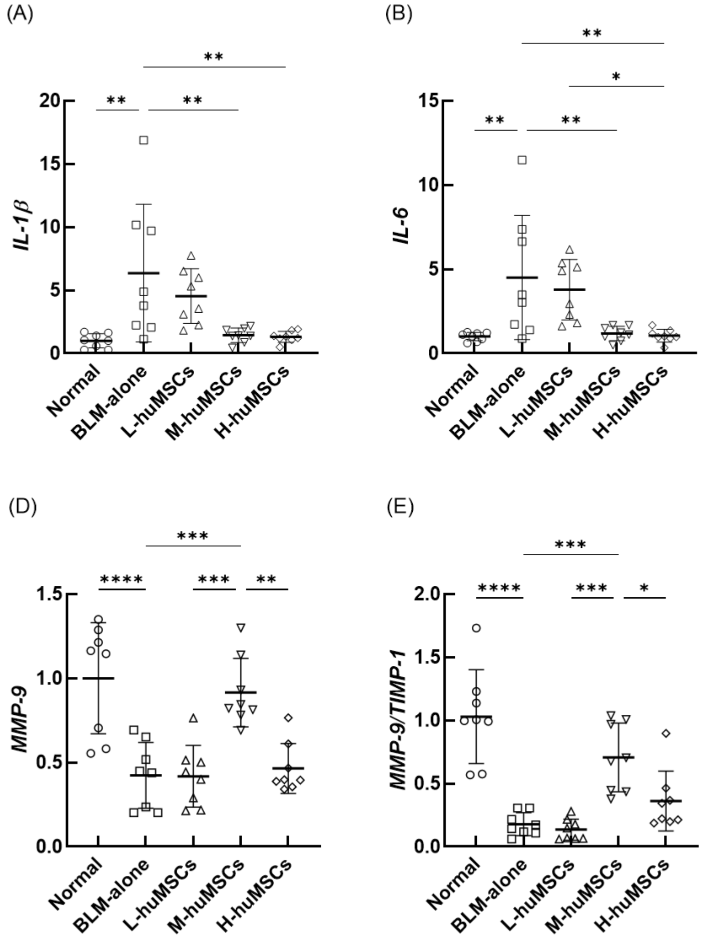

Figure S2. Quantitative reverse transcription-PCR analysis of pulmonary mRNA expression. Quantitative reverse transcription-PCR analysis of lung mRNA expression. The relative mRNA expression of IL-1β **(A),** IL-6 **(B),** TIMP-1 **(C),** MMP-9 **(D)**, and MMP-9/TIMP-1 **(E)** were evaluated in the lung tissues of BLM-induced ILD mice at day 28. Experimental groups included: Normal (untreated control mice), BLM-alone (mice with ILD induced by bleomycin), L-huMSCs (low-dose huMSCs, 1×10^3 cells), M-huMSCs (medium-dose huMSCs, 1×10^4 cells), and H-huMSCs (high-dose huMSCs, 1×10^5 cells). Results are presented as the mean ± SD (N = 8 mice per group). Statistical significance is denoted as follows: * $p < 0.05$, ** $p < 0.01$, *** $p < 0.001$, **** $p < 0.0001$, with significant differences highlighted between the indicated groups.

Table S1. Primers used in the qPCR analyses in vitro

Gene	Forward (5' to 3')	Reverse (5' to 3')
CD36	TCAGGACCCCGAGGACCACAC	AGGAGGCTGCGTCTGTGCCA
CD163	TCACTCCTGGGCTGCACGTAAAC	GATGTTATTTGCCATACAGGAGAATTG
TNF-α	ACCTTGTTGCCTCCTCTT	GTTCAGTGATGTAGCGACAG
IL-10	GTGGAGCAGGTGAAGAGTGA	TTCATGGCCTTGTAGACACCT
GAPDH	ACAATGAATACGGCTACAG	GGTCCAGGGTTTCTTACT

Table S2. Primers used in the qPCR analyses in vivo

Gene	Forward (5' to 3')	Reverse (5' to 3')
IL-6	AAATGAGAAAAGAGTTGTG	TTTGTATCTCTGGAAGTTT
IL-1β	GATACCACTCCCAACAGA	GCCATTGCACAACTCTTT
MMP-9	CGATTCCAAACCTTCAAA	GCAAGTCTTCAGAGTAGT
TIMP-1	AAGATGACTAAGATGCTAA	GATGAGAAACTCTTCACT
GAPDH	ACAATGAATACGGCTACAG	GGTCCAGGGTTTCTTACT
